# Tyrosine‐protein phosphatase non‐receptor type 2 inhibits alveolar bone resorption in diabetic periodontitis via dephosphorylating CSF1 receptor

**DOI:** 10.1111/jcmm.14545

**Published:** 2019-08-02

**Authors:** Dongjiao Zhang, Yanfei Jiang, Dawei Song, Zhenkun Zhu, Cong Zhou, Li Dai, Xin Xu

**Affiliations:** ^1^ Shandong Provincial Key Laboratory of Oral Tissue Regeneration, School of Stomatology Shandong University Jinan China; ^2^ Department of Implantology, School of Stomatology Shandong University Jinan China; ^3^ Beijing Tsinghua Changgung Hospital, Tsinghua University Beijing China; ^4^ The Seventh People's Hospital of Shenzhen Shenzhen China

**Keywords:** 25(OH)2D_3_, alveolar bone resorption, CSF1R, diabetic periodontitis, tyrosine‐protein phosphatase non‐receptor type 2

## Abstract

Tyrosine‐protein phosphatase non‐receptor type 2 (PTPN2) is an important protection factor for diabetes and periodontitis, but the underlying mechanism remains elusive. This study aimed to identify the substrate of PTPN2 in mediating beneficial effects of 25‐Hydroxyvitamin D_3_ (25(OH)2D_3_) on diabetic periodontitis. 25(OH)2D_3_ photo‐affinity probe was synthesized with the minimalist linker and its efficacy to inhibit alveolar bone loss, and inflammation was evaluated in diabetic periodontitis mice. The probe was used to pull down the lysates of primary gingival fibroblasts. We identified PTPN2 as a direct target of 25(OH)2D_3_, which effectively inhibited inflammation and bone resorption in diabetic periodontitis mice. In addition, we found that colony‐stimulating factor 1 receptor (CSF1R) rather than JAK/STAT was the substrate of PTPN2 to regulate bone resorption. PTPN2 direct interacted with CSF1R and dephosphorylated Tyr807 residue. In conclusion, PTPN2 dephosphorylates CSF1R at Y807 site and inhibits alveolar bone resorption in diabetic periodontitis mice. PTPN2 and CSF1R are potential targets for the therapy of diabetic periodontitis or other bone loss‐related diseases.

## 1 INTRODUCTION

Periodontitis involves progressive loss of the alveolar bone around the teeth, which not only affects oral health but also aggravates such systemic diseases as cardiovascular, respiratory illnesses and diabetes.^1^, ^2^, ^3^, ^4^ Poorly controlled diabetes in turn increase the susceptibility to periodontitis, which is called diabetic periodontitis.^5^, ^6^ Compared with non‐diabetic patients, diabetic patients exhibit a poorer prognosis and worse alveolar bone loss.^7^ Therefore, exploring the mechanisms underlying bone loss in diabetic periodontitis is of great importance to guide clinical therapy.

Vitamin D regulates immunity and bone metabolism and plays a significant role in calcium metabolism and homoeostasis.^8^ 25‐Hydroxyvitamin D_3_ (25(OH)2D_3_) is the stable condition of VD_3_ in the body.^9^, ^10^ Previous studies have shown that 25(OH)2D_3_ supplement decreased inflammation factor TNF‐α in monocytes and inhibited the proliferation of T cells.^10^, ^11^ Moreover, 25(OH)2D_3_ ameliorated experimental periodontitis in diabetic mice.^12^ While it is known that 25(OH)2D_3_ binds to vitamin D receptor (VDR) to mediates its biological effects, the detailed mechanism of action needs further investigations.

Tyrosine‐protein phosphatase non‐receptor type 2 is essential to bone homoeostasis and development because the number of osteoclast and bone resorption increase in PTPN2 knockout mice.^13^ PTPN2 is a negative regulator of many signalling pathways by dephosphorylating the corresponding substrates, including non‐receptor protein tyrosine kinases, receptor protein tyrosine kinases and the Src family kinases, either in the nucleus or in the cytoplasm.^14^, ^15^, ^16^, ^17^ Among the signalling molecules, receptor activator for nuclear factor‐κ B Ligand (RANKL) and colony‐stimulating factor‐1 (CSF‐1) play an important role in osteoclastogenesis.^18^, ^19^ CSF‐1 binds to the unique receptor tyrosine kinase CSF1R on osteoclasts to activate signalling cascade and induce the expression of osteoclast lineage‐related genes, including calcitonin receptor, 3‐integrin 7, cathepsin K (CATK) and tartrate‐resistant acid phosphatase (TRAP).^20^, ^21^ Interestingly, PTPN2 could negatively regulate CSF1R signalling and mononuclear phagocyte development in hematopoiesis.^22^ However, it remains unclear whether PTPN2 regulates CSF‐1/CSF1R signalling in osteoclastogenesis.

Therefore, in present study, we aimed to investigate the crosstalk of PTPN2 and CSF‐1/CSF1R signalling in the regulation of alveolar bone resorption in the condition of diabetic periodontitis.

## 2 MATERIALS AND METHODS

### 2.1 Antibodies

The antibodies used were as follows: PTPN2, STAT1, STAT3 and p‐p38 (1:1000 dilution) (Santa Cruz Biotechnology), p38, p‐STAT1, p‐STAT3, CSF1R and p‐CSF1R (1:1000 dilution, Abcam), Biotin, Flag (1:10 000 dilution) (Sigma), and β‐actin (1:3000 dilution) (Cell Signalling). The anti‐mouse CSF1R peptide (^962^GDIAQPLLQPNNYQF^976^) antiserum (CT) and the anti‐pY559‐CSF1R peptide antibody (^555^EGNSpYTFIDPTQLPYNEK^572^) were raised in rabbits and affinity purified against their corresponding peptides.^23^

### 2.2 Mutant constructs

CSF‐1 Y807F (Tyr at 807 residue changed to phenylalanine) was constricted using QuikChange Site‐Directed Mutagenesis kit (Stratagene) according to the manufacturer's protocol. PTPN2‐MT (D182A) (Asp at 182 residue changed to Ala) had reduced catalytic activity but same affinity to the substrate compared with wild‐type PTPN2.^24^ PTPN2 D182A mutant was constructed using QuikChange Site‐Directed Mutagenesis kit (Stratagene) according to the manufacturer's protocol. All mutant constructs were validated by DNA sequencing.

### 2.3 Animals

Four‐week‐old male C57BL/6 wild‐type mice were purchased from the Experimental Animal Laboratory of Shandong University and maintained in the cage individually with an artificial light cycle at room temperature (23 ± 2℃) and humidity (55 ± 5%). Mice were given free access to food and tap water. All of the experiments were performed in accordance with Guide for the Care and Use of Laboratory Animals of the National Research Council (the USA, 2011) and approved by the IRB of Shandong University (No. GD201608).

At 6‐week old, the mice consumed high‐fat food (48 kcal% fat) for 7 days and fasted overnight. Then the mice received intraperitoneal injection of streptozotocin (STZ, Sigma, dissolved in citric acid buffer, pH 4.5) for five consecutive days at a dose of 40 mg/kg (bodyweight). After STZ injection the mice continued high‐fat food.

To induce periodontitis, the mice were infected with *Porphyromonas gingivalis* W50 (ATCC: 53978) at the age of 8 weeks as described previously.^25^ Control mice received 100 μL of PBS with 2% carboxymethylcellulose.

25(OH)2D_3_‐probe was synthesized as reported previously.^26^ 25(OH)2D_3_ or 25(OH)2D_3_‐Probe was dissolved in refined peanut oil, and mice received intraperitoneal injection of 25(OH)2D_3_ or 25(OH)2D_3_‐Probe every 2 days from the age of 8 weeks at the dose of 5 μg/kg bodyweight.

### 2.4 Bodyweight and fasting blood glucose measurement

The body weight and fasting blood glucose of the mice in each group were monitored every 2 weeks. Briefly, after fasting for 10 hours, the tail veins of the mice were cut, and the blood was collected, and fasting blood glucose levels were measured using a glucometer (OneTouch Glucometer).

### 2.5 Stereotaxic injection of virus

On the fourth day after the infection with *Porphyromonas gingivalis,* the mice were anesthetized by intraperitoneal injection of 5% chloral hydrate. 2 × 10^9^ adeno‐associated virus (AAV) shPTPN2 viral particles (Santa Cruz Biotechnology) was injected about 0.3‐0.5 mm above the gingival margin of the maxillary molars on the right and left palatal aspects. The injection was repeated for seven consecutive days.

### 2.6 ELISA

CO_2_ inhalation was used to sacrifice the animals on week 18 after the initial infection. 100‐150 μL of blood was collected from the punctured heart. TNF‐α, IL‐6 and IFN‐γ levels in the serum were determined by ELISA kits (Life Technologies) according to the manufacturer's protocol.

### 2.7 Histological analysis

After the sacrifice, the mandibular jaws were gathered and boiled in water for 15 minutes to remove the soft tissues. The alveolar bone loss of the second molar was assessed by a stereomicroscope (×15) with an attached digital camera (Leica MZ FLIII, Germany). Next, the maxillary jaws were fixed by 4% paraformaldehyde for 24 hours and then decalcified by 10% EDTA for 25 days. The samples were dehydrated using 50%, 70%, 95% and 100% alcohol, embedded in paraffin and then cut into 4‐μm sections for haematoxylin‐eosin (H&E) staining.

### 2.8 Cell isolation and culture

Primary gingival fibroblasts were isolated from mandibular tissues obtained from diabetic periodontitis mice. Briefly, the gingival tissue was rinsed six times in PBS to diminish microbial contaminants and then finely minced with scalpels in a 35‐mm dish (Corning, Corning Incorporated) containing 2 mL of DMEM supplemented with 10% foetal bovine serum and 1% penicillin/streptomycin. The gingival fibroblasts were allowed to explant from the minced tissue in complete medium with changes every 3 days. The cells were seeded at a density 2 × 10^4^ cells/cm^2^ and cultured to 80% confluency for the following experiments.

### 2.9 Western blot analysis

The cells or tissue were lysed in lysis buffer (150 mmol/L NaCl, 20 mmol/L Tris‐HCl, 10% glycerol, 1% Triton X‐100, 1 mmol/L Na_3_VO_4_, 0.1 mmol/L PMSF, pH 7.4) on ice for 20 minutes and centrifuged at 20 000 × g for 20 minutes. The supernatants were subjected to Western blot analysis using appropriate primary and secondary antibodies for xx.

### 2.10 Pull‐down analysis

The lysates of primary gingival cells were mixed with 50 μmol/L Biotin‐N_3_ and 25(OH)2D_3_‐Probe or 25(OH)2D_3_. Then 2 mmol/L TCEP, 2 mmol/L CuSO_4_ and 200 μmol/L TBTA were added to the lysates and incubated with streptavidin agarose (Invitrogen) at 4°C overnight. On the following day, the beads were washed six times with lysis buffer on ice after UV irradiation for 20 minutes, then the immunoprecipitations were eluted using low pH elution buffer at 4°C for 15 minutes. 1/20 volume of 1 mol/L Tris‐HCl (pH 9.4) was used to neutralize the acid elution, and the eluted bead‐bound proteins were separated by SDS‐PAGE and detected by silver staining or Western blot analysis.

### 2.11 Immunofluorescence

Primary gingival cells were grown to 50%‐70% confluence and treated with 400 ng/mL 25(OH)2D_3_ or DMSO for 24 hours. After washing with PBS, the cells were fixed for 10 minutes in freshly prepared 4% paraformaldehyde and then permeabilized for 10 minutes in 0.25% Triton X‐100. The cells were blocked for 1 hour with 2 mg/mL BSA at room temperature and then incubated with antibody for PTPN2‐45kD or CSF1R at 4°C overnight. The cells were then incubated with appropriate secondary antibody for 1 hour at room temperature in the dark. Finally, the nuclei were stained by DAPI at 37°C for 30 minutes in the dark. The stained cells were observed using confocal microscope system (CSU10, Yokogawa Electric Co) with an inverted microscope (IX‐71, Olympus Optical Co., Ltd) and a CoolSNAP‐HQ camera (Roper Industries).

### 2.12 Statistical analysis

Data were expressed as mean ± SD and analysed using SPSS 12.0 statistical analysis package (SPSS Inc). The comparison was analysed using t test or ANOVA *P* < .05 was accepted as significant difference.

## 3 RESULTS

### 3.1 **25(OH)2D_3_**‐**probe inhibited alveolar bone loss and inflammation**

**Figure 1:**
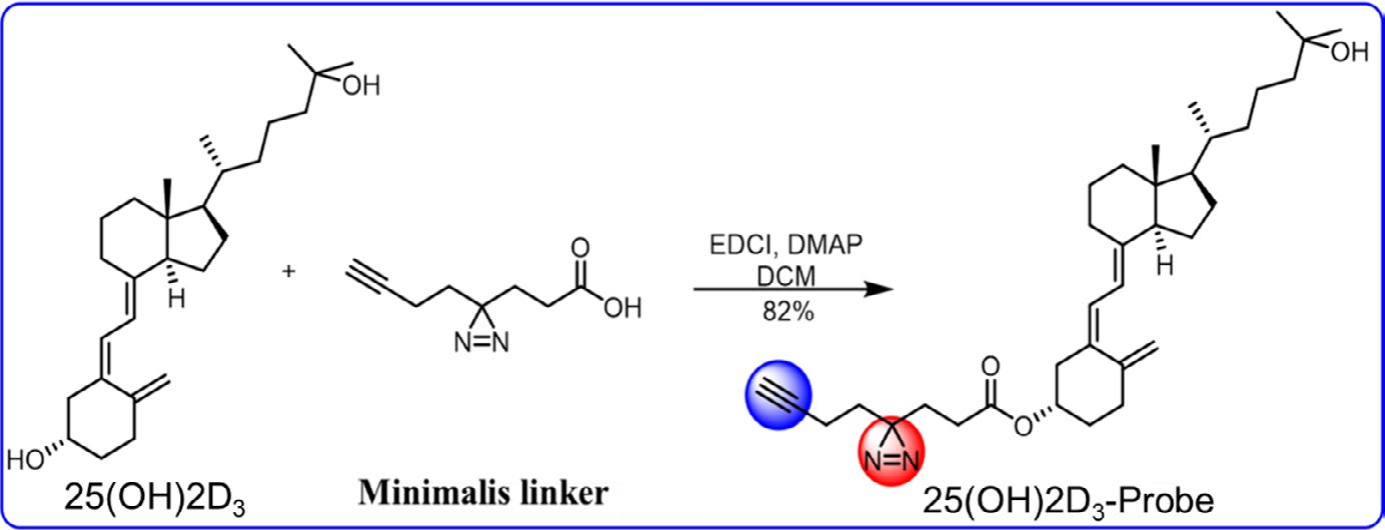
Synthesis of 25(OH)2D_3_‐Probe

**Figure 2:**
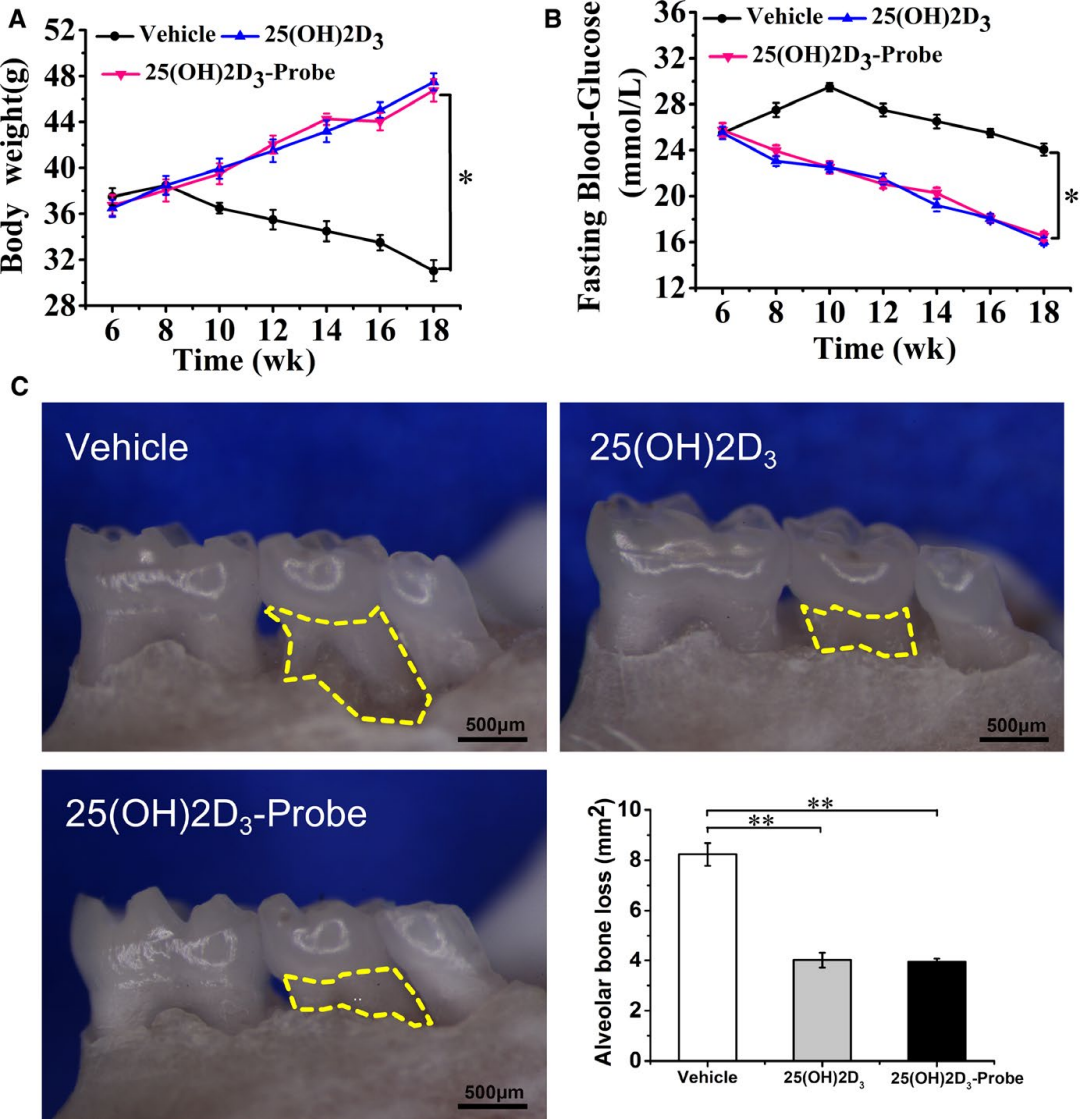
25 VD_3_ ‐Probe reduced alveolar bone loss. Diabetic periodontitis models were induced, and 25(OH)2D_3_ or 25(OH)2D_3_‐probe was intraperitoneally injected. A, Bodyweight; B, fasting glucose were monitored every 2 d until sacrifice. C, Alveolar bone resorption area of the second maxilla molars was measured under the stereoscopic microscope. Yellow‐dotted areas indicate horizontal bone resorption. Data are mean ± SD (n = 8). *P < .05, **P < .01 compared to vehicle group

To identify functional target proteins of 25(OH)2D_3_, we first synthesized the photo‐affinity probe by modifying 25(OH)2D_3_ with the minimalist linker, incorporating both an alkyl diazirine and a terminal alkyne in an aliphatic chain (Figure 1). To confirm that the probe keeps biological activities of 25(OH)2D_3_, we compared the effects of the probe and 25(OH)2D_3_ in mouse model of diabetic periodontitis. Compared with vehicle group, intraperitoneal injection of 25(OH)2D_3_ or 25(OH)2D_3_‐Probe significantly increased bodyweight, decreased fasting blood glucose and inhibited alveolar bone loss, but there were no significant differences between 25(OH)2D_3_ and 25(OH)2D_3_‐Probe groups (Figure 2A‐C). In addition, serum levels of TNFα, IFN‐γ and IL‐6 were significantly lower in 25(OH)2D_3_ and 25(OH)2D_3_‐Probe groups than in vehicle control group. Serum levels of IFN‐γ and IL‐6 showed no significant difference between 25(OH)2D_3_ and 25(OH)2D_3_‐Probe groups although serum TNFα level was lower in 25(OH)2D_3_ group than in 25(OH)2D_3_‐Probe group (Table 1). Collectively, these data indicate that 25(OH)2D_3_ probe did not compromise biological activity of 25(OH)2D_3_, and it is suitable for target identification.

**Table 1:** Serum cytokine levels in mouse treated with 25(OH)2D_3_

Group	TNFα (pg/mL)	IFN‐γ (pg/mL)	IL‐6 (pg/mL)
Vehicle	57.74 ± 1.31	213.56 ± 7.48	7.29 ± 0.19
25(OH)2D_3_	26.63 ± 2.01**	102.32 ± 1.90**	4.06 ± 0.61*
25(OH)2D_3_‐Probe	44.35 ± 5.37*,***	114.33 ± 10.56**	5.03 ± 0.52*

Note

Data are mean ± SD (n = 8).

* *P* < .05

** *P *< .01 compared to control (Vehicle).

*** *P* < .05 for 25(OH)2D_3_‐Probe VS 25(OH)2D_3_.

### 3.2 25(OH)2D_3_ stimulates PTPN2 phosphatase activity

**Figure 3:**
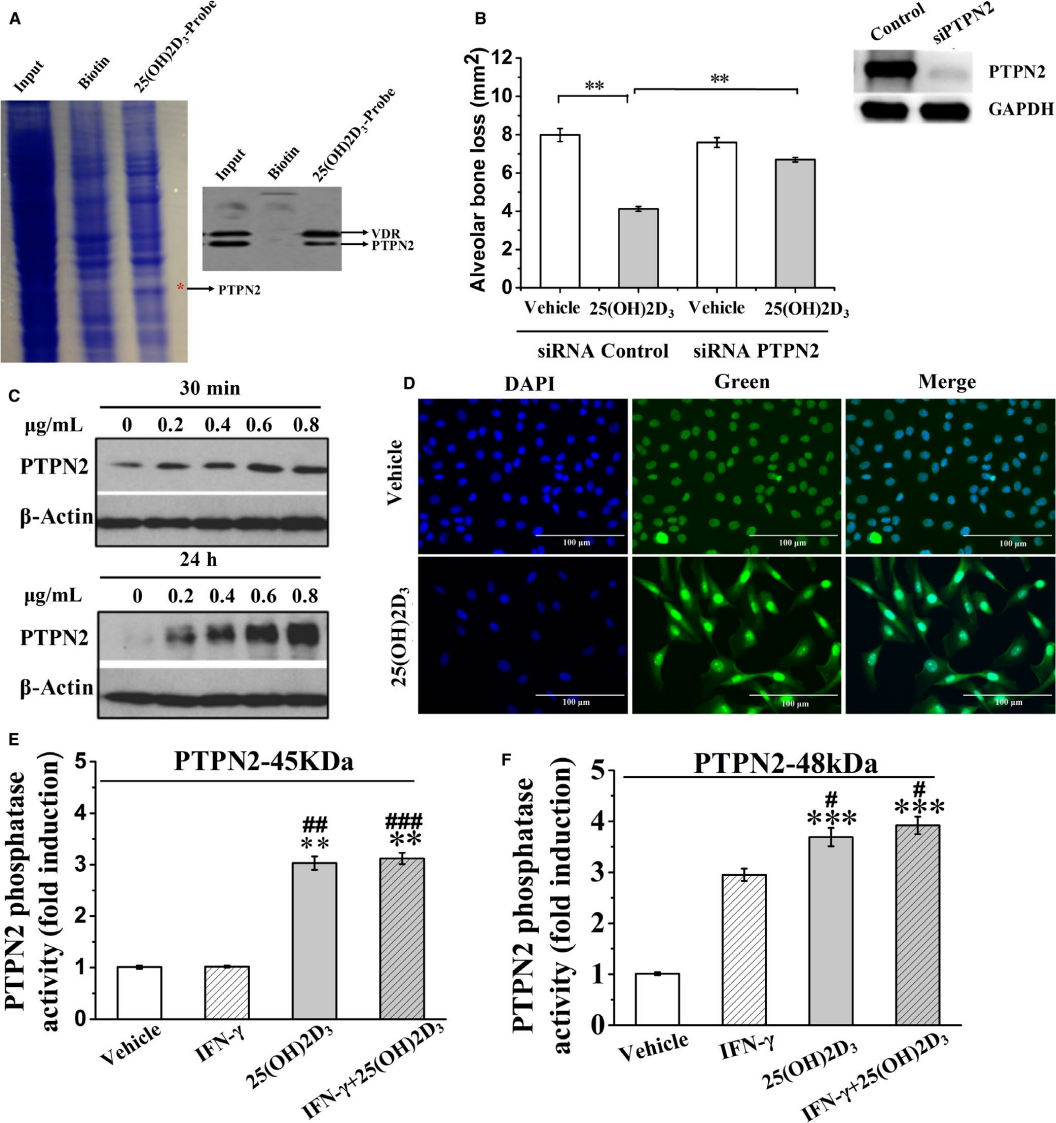
25(OH)2D_3_ induces PTPN2 phosphatase activity. A, Primary gingival fibroblast lysates were incubated with Biotin or 25(OH)2D_3_‐probe and then exposed to UV, after pull‐down the precipitates were resolved in SDS‐PAGE and stained by Coomassie Blue. The indicated bands were examined by mass spectrometry and Western blot analysis. B, AAV vector encoding PTPN2 shRNA was injected into the gingival tissue of diabetic periodontitis mice, which were then treated with 25(OH)2D_3_ and alveolar bone loss was evaluated. C, Primary gingival fibroblasts were treated with 25(OH)2D_3_, and PTPN2 protein expression level was analysed by Western blot. D, Primary gingival fibroblasts were treated with 25(OH)2D_3_, stained by PTPN2 antibody (green), and then counterstained with DAPI (blue). E‐F, Primary gingival fibroblasts were treated with 25(OH)2D_3_, and phosphatase activity of PTPN2‐45kDa was measured. Data are mean ± SD (n = 8). **P < .01, ***P < .001 compared to vehicle group. # *P *< .05, ##*P *< .01, ###*P *< .001 compared to IFN‐γ group

Using 25(OH)2D_3_‐Probe and lysates of primary gingival fibroblasts, we performed affinity purification and identified PTPN2 as the target protein of 25(OH)2D_3_ (Figure 3A). To reveal the function of PTPN2 in mediating the effects of 25(OH)2D_3_, we used AAV‐PTPN2 shRNA to knock‐down PTPN2 in gingival fibroblasts of the mice with diabetic periodontitis. The ability of 25(OH)2D_3_ to alleviate inflammation and protect alveolar bone loss was almost completely abolished in mice injected with AAV‐PTPN2 shRNA (Table 2, Figure 3B). These results strongly suggest that 25(OH)2D_3_ targets PTPN2 to improve diabetic periodontitis.

**Table 2:** Serum cytokine levels in mouse treated with PTPN2 shRNA and 25(OH)2D_3_

Group	TNFα (pg/mL)	IFN‐γ (pg/mL)	IL‐6 (pg/mL)
shRNA Control			
Vehicle 25(OH)2D_3_	58.24 ± 2.30	215.19 ± 13.27	6.04 ± 0.12
	30.02 ± 0.27*	113.56 ± 7.41**	4.12 ± 0.16*
shRNA PTPN2			
Vehicle 25(OH)2D_3_	124.67 ± 2.08	312.68 ± 2.15	5.94 ± 0.33
	112.04 ± 0.27	299.89 ± 5.34	5.62 ± 0.82

Note

Data are mean ± SD (n = 8).

* *P *< .05.

** *P *< .01 compared to control (Vehicle).

To elucidate the mechanism by which PTPN2 mediates biological effects of 25(OH)2D_3_, we treated primary gingival fibroblasts with 25(OH)2D_3_ and observed increased expression of PTPN2 (Figure 3C). In addition, 25(OH)2D_3_ stimulated the translocation of PTPN2‐45kDa from the nuclei into cytoplasm (Figure 3D). Next, we purified PTPN2‐45kDa by immunoprecipitation and examined its phosphatase activity. Compared to cells treated with vehicle or IFN‐γ alone, PTPN2‐45kDa phosphatase activity increased significantly after treatment with 25(OH)2D_3_ or with IFN‐γ and 25(OH)2D_3_ for 30 minutes (*P* < .01 and *P* < .001, respectively) (Figure 3E,F). These results indicate that 25(OH)2D_3_ targets PTPN2 and increases its phosphatase activity.

### 3.3 CSF1R is the substrate of PTPN2

**Figure 4:**
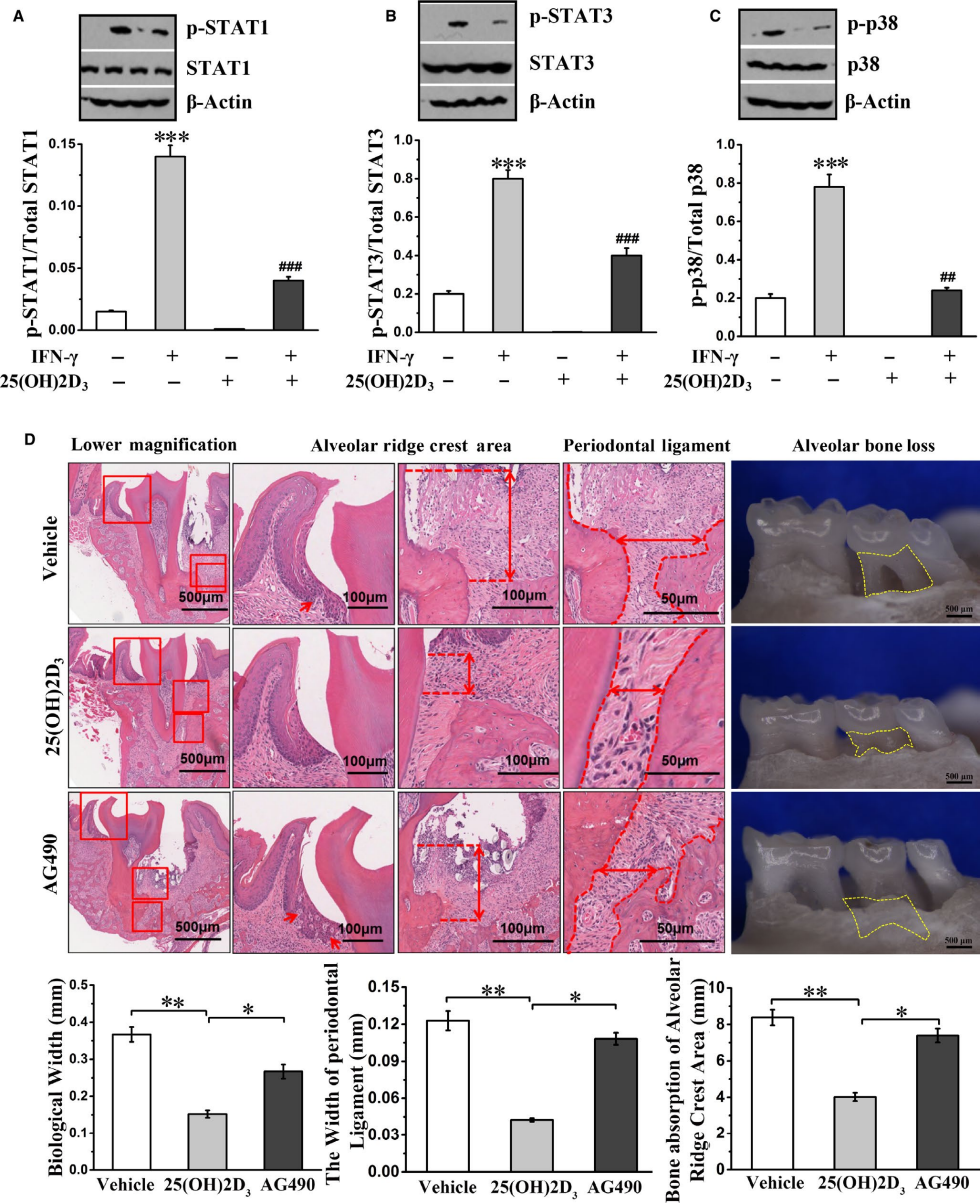
25(OH)2D_3_ inhibits the phosphorylation of STAT1, STAT3 and p38, but STAT inhibitor has no effect on bone resorption. A‐C, Primary gingival fibroblasts were incubated with IFN‐γ and/or 25(OH)2D_3_, and the phosphorylation of STAT1, STAT3 and p38 was detected by Western blot. D, Periodontal tissue and the mandibular molar of diabetic periodontitis mice were stained with H&E and then observed by the stereomicroscope. Red arrows represent the epithelial spikes. Yellow‐dotted areas indicate horizontal alveolar bone loss. Data are mean ± SD, n = 8. *P < .05, ** *P *< .01, ***P < .001 compared to without IFN‐γ and 25(OH)2D_3_ group or vehicle group; ##*P *< .01, ### *P *< .001 compared to 25(OH)2D_3_ group

Since PTPN2 inhibits inflammation by negatively regulating classic inflammatory JAK/STAT pathway, we examined whether anti‐inflammation activity of 25(OH)2D_3_ is mediated via negative regulation of JAK/STAT pathway by PTPN2. As shown in Fig. 4A‐C, 25(OH)2D_3_‐mediated PTPN2 activation inhibited the phosphorylation of STAT1, STAT3 and p38. However, classic Akt/STAT inhibitor AG‐490 did not exhibit the same protective effect on bone resorption as 25(OH)2D_3_, implying that some unidentified PTPN2 substrates may be responsible for the effects of 25(OH)2D_3_ on bone resorption (Figure 4D).

**Figure 5:**
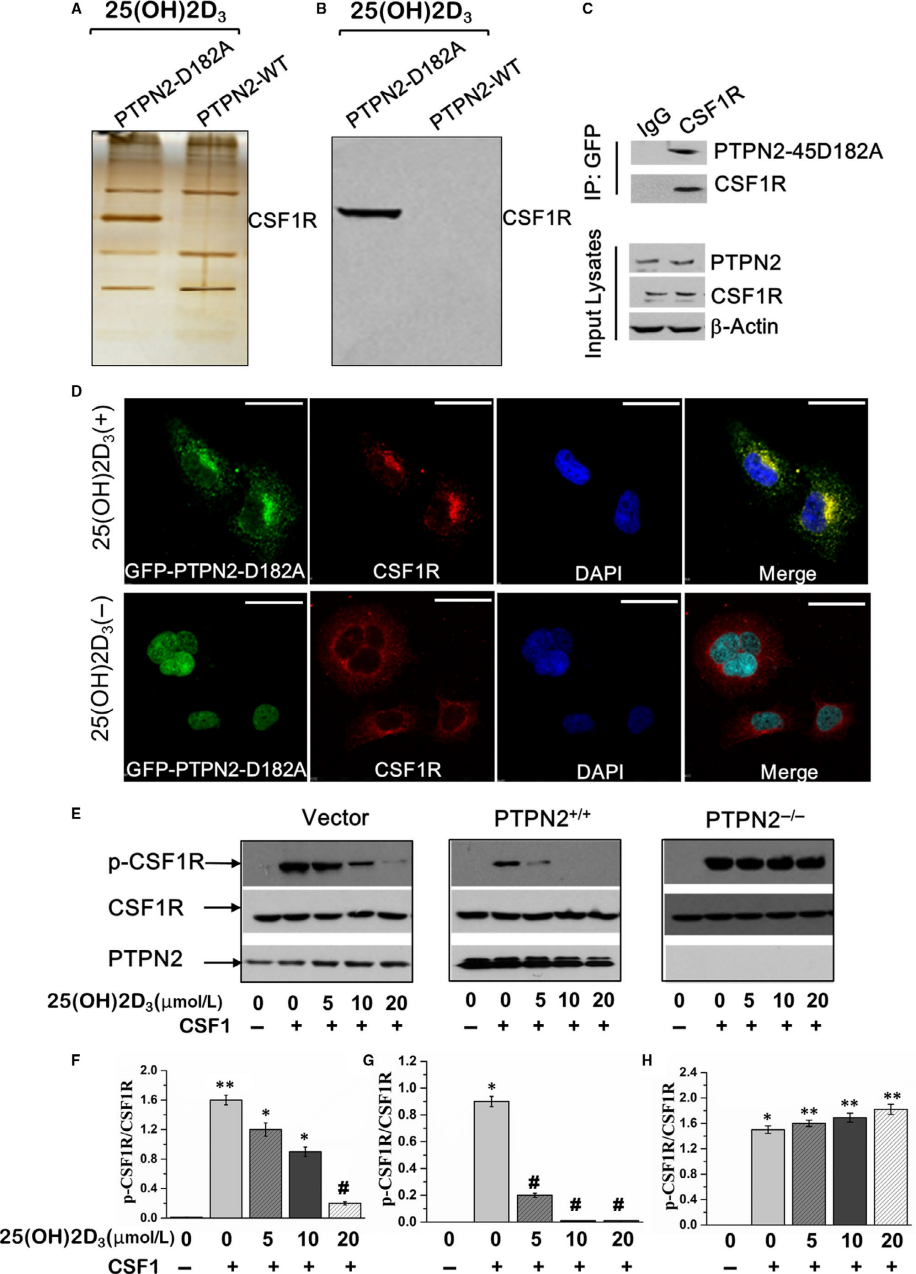
PTPN2 dephosphorylates CSF1R. Primary gingival fibroblasts were transfected with PTPN2‐45D182A mutant or PTPN2‐WT and treated with 25(OH)2D_3_. Cell lysates were subjected to (A) silver staining and (B) Western blot. C, GST pull‐down assay of HEK293T cells transfected with GST‐PTPN2‐45D182A and HA‐CSF1R. D, Immunofluorescence microscopy of gingival fibroblasts transfected with GFP‐tagged PTPN2‐45D182A (green), CSF1R was stained by antibody (red) and the nuclei stained by DAPI (blue), Scar bar, 20 μm. (E‐H) Primary gingival fibroblasts were transfected with vector, WT‐PTPN2, or siPTPN2, and then treated with 25(OH)2D_3_ or/and CSF1. The phosphorylated CSF1R was analysed by Western blot. Data are mean ± SD. *P < .05, **P < .01, compared to vehicle group; ##*P *< .01 compared to CSF1 group

To identify the substrates of PTPN2‐45kDa in the presence of 25(OH)2D_3_, we transfected primary gingival fibroblasts with either PTPN2‐WT or PTPN2‐45D182A construct and then stimulated the cells with 25(OH)2D_3_. The bands only appeared with PTPN2‐45D1892A but not PTPN2‐WT were cut down, and CSF1R was identified (Figure 5A). Western blot analysis confirmed the band as CSF1R (Figure 5B). In addition, glutathione S‐transferase (GST) pull‐down assay verified the interaction of PTPN2‐45D182A with CSF1R (Figure 5C). Furthermore, we transfected gingival fibroblasts with GFP‐tagged PTPN2‐45D182A. GFP‐tagged PTPN2‐45D182A appeared in the nuclei in basic condition, after 25(OH)2D_3_ stimulation it appeared in the cytoplasm and was co‐localized with CSF1R (Figure 5D). To determine whether PTPN2 could directly dephosphorylate CSF1R, we detected the phosphorylation status of CSF1R by altering PTPN2 expression level. PTPN2 overexpression or 25(OH)2D_3_ stimulation led to significantly decreased and shortened phosphorylation of CSF1R, while knock‐down of PTPN2 led to strong and constitutive phosphorylation of CSF1R (Figure 5E). Taken together, these results confirm that PTPN2 directly dephosphorylates CSF1R.

### 3.4 Tyrosine 807 of CSF1R is the main site for the dephosphorylation by PTPN2

**Figure 6:**
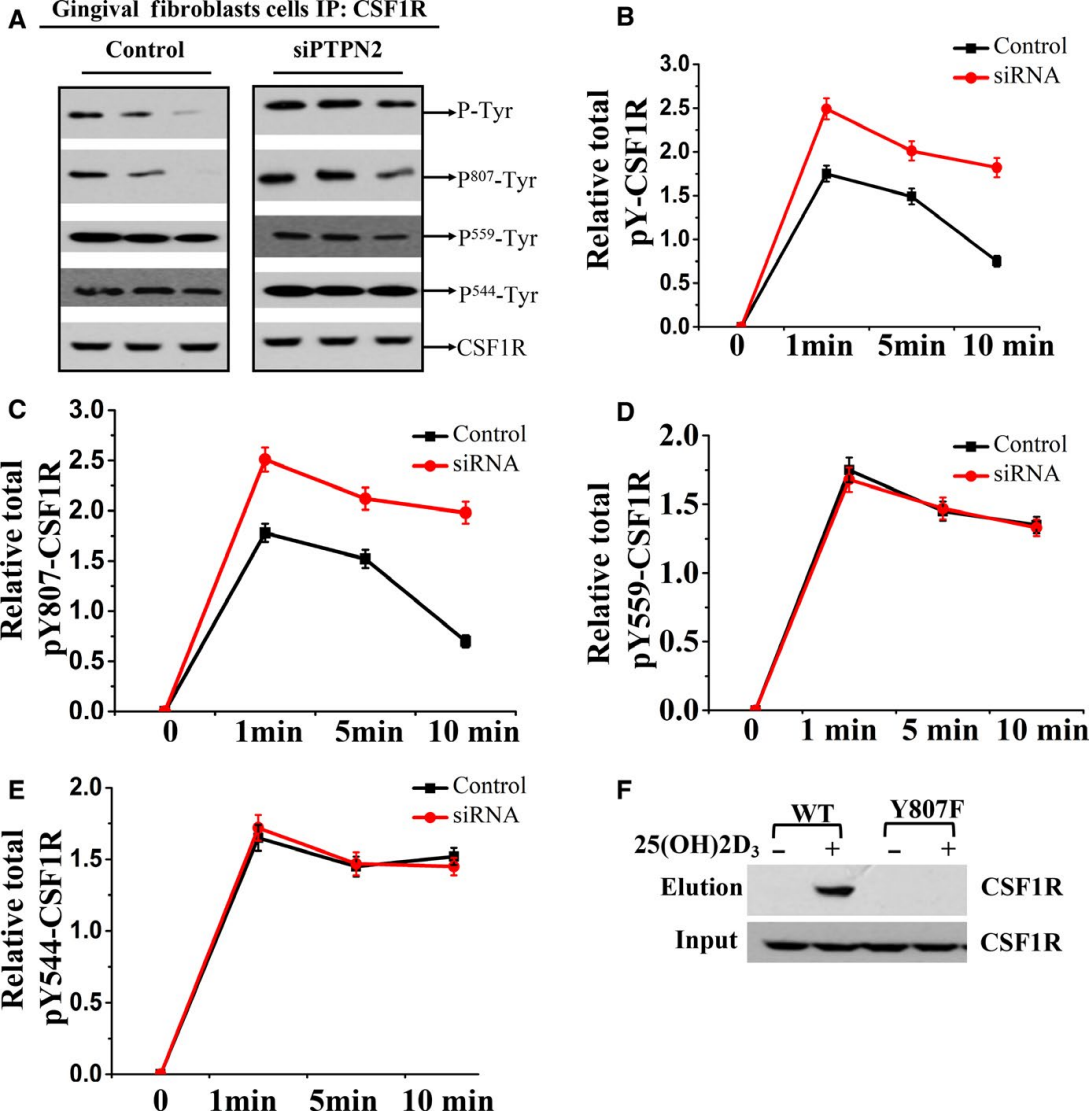
Y807 is the main site on CSF1R dephosphorylated by PTPN2. (A) Primary gingival fibroblasts isolated from diabetic periodontitis mice were transfected with Control siRNA and PTPN2 siRNA and stimulated with M‐CSF, and the cell lysates were immunoblotted with antibody recognizing p‐Tyr, p^807^‐Tyr, P^559^‐Tyr, p^544^‐Tyr, CSF1R. Quantification of the phosphorylation level of (B) CSF1R, (C) Y807‐CSF1R, (D) Y559‐CSF1R and (E) Y544‐CSF1R. F, HEK293T cells were co‐transfected with PTPN2‐45D182A plasmid and either WT‐CSF1R or Y807F‐CSF1R. Transfected cells were stimulated with 25(OH)2D_3_ and the interaction of CSF1R with PTPN2‐45D182A was analysed by Western blot analysis

Next, we explored the specific tyrosine sites on CSF1R that are the targets of PTPN2. Upon CSF1 ligand binding, CSF‐1R extracellular domain dimerizes and induces the phosphorylation of six phosphorylation sites in cytoplasmic domain, tyrosine 559, 697, 706, 721, 807 and 974. PTPN2 knock‐down led to enhanced and prolonged overall tyrosine phosphorylation of CSF1R, but had no significant effects on auto‐phosphorylation of Tyr544 and Tyr559 (Figure 6A‐E). In HEK293T cells transfected with wild‐type or PTPN2‐45D182A mutant or mutant Y807F‐CSF1R constructs, 25(OH)2D_3_ stimulation increased the association of PTPN2‐45D182A with WT‐CSF1R but not Y807F‐CSF1R (Figure 6F). Collectively, these results indicate that Y807 at CSF1R is the main site being dephosphorylated by PTPN2 in response to 25(OH)2D_3_.

## 4 DISCUSSION

In this study, we employed a variety of approaches to elucidate novel mechanism of the action of 25(OH)2D_3_ on periodontitis and made important findings as follows: first, we synthesized photo‐affinity probe of 25(OH)2D_3_, which effectively reduced alveolar bone loss and inhibited inflammatory cytokines secretion in mice with diabetic periodontitis. Second, we identified PTPN2‐45kD as a target of 25(OH)2D_3_ and found that PTPN2 mediated the effects of 25(OH)2D_3_ on alveolar bone loss not through JAK/STAT pathway. Third, we demonstrated that PTPN2 directly dephosphorylated its substrate CSF1R on Y807 site.

Clinical and epidemiological data suggest that people with diabetes are susceptible to periodontitis, which indicates that diabetes is a considerable risk factor for periodontitis.^27^, ^28^, ^29^ 25(OH)2D_3_ has been proven to reduce inflammation and bone resorption in periodontitis, but the molecular mechanisms need further investigation.

Using photo‐affinity probe of small molecules to explore target protein is by far the most commonly used method to reveal the mechanism of action. In this study, we successfully synthesized 25(OH)2D_3_ photo‐affinity probe by modifying 25(OH)2D_3_ with the minimalist linker and demonstrated that it had similar effect as 25(OH)2D_3_ in inhibiting bone loss and inflammatory response in diabetic periodontitis mice. Therefore, we used it to identify target proteins that mediate beneficial effects of 25(OH)2D_3_ on diabetic periodontitis.

We performed affinity pull‐down analysis and identified that 25(OH)2D_3_ could directly bind PTPN2. There exist two forms of PTPN2: a nuclear 45‐kDa form (PTPN2‐45kDa) and an endoplasmic reticulum targeted 48‐kDa form (PTPN2‐48kDa). In response to external stimuli, PTPN2‐45kDa translocates from the nuclei to the cytoplasm and plasma membrane, where it can dephosphorylate its substrates and regulate related signalling pathways.^30^ We found that PTPN2 expression increased after stimulation by 25(OH)2D_3_, and PTPN2‐45kD quickly translocated into the cytoplasm. In addition, PTPN2‐45kDa phosphatase activity increased significantly after treatment with 25(OH)2D_3_. Therefore, 25(OH)2D_3_ causes nuclear exit of PTPN2‐45kDa and increases its enzymatic activity.

Tyrosine‐protein phosphatase non‐receptor type 2 regulates various cellular signalling pathways and biological processes by dephosphorylating its physiological substrates. JAK/STAT signalling pathway is one of known inflammation‐related pathways that is negatively regulated by PTPN2.^30^ In addition, 25(OH)2D_3_ has been reported to show therapeutic effect in diabetic periodontitis mice through regulation JAK1/STAT3 signalling.^12^ Consistent with these reports, we found that 25(OH)2D_3_‐induced PTPN2 activation inhibited the phosphorylation of STAT1, STAT3 and p38, which may mediate anti‐inflammation activity of 25(OH)2D_3_ in vivo. However, when we used classic STAT inhibitor AG‐490 to inhibit JAK1/STAT3 signalling, we did not observe the same protective effect on bone resorption as with 25(OH)2D_3_, indicating that additional substrates other than JAK1/STAT3 are responsible to inhibit bone resorption downstream of 25(OH)2D_3_ and PTPN2.

PTPN2‐D182A mutant has less enzyme activity but maintains the affinity to the substrates, thus can form stable complex with the substrates. To this end, we generated substrate‐trapping mutant PTPN2‐45D182A. We showed that PTPN2 substrate‐trapping mutant interacted with CSF1R in the presence of the stimulus of 25(OH)2D_3_. Tyrosine 544, 559 and 807 on CSF1R are required for the regulation of macrophage proliferation.^23^ To identify which tyrosine residues on CSF1R are the targeting sites of PTPN2, we detected the phosphorylation status of these residues by using the particular phosphorylation antibodies and knock‐down of PTPN2. Our results indicate that Y807 residue of CSF1R is the main site to be dephosphorylated by PTPN2 in response to 25(OH)2D_3_. Interestingly, CSF1R was reported to regulate osteoclast formation and function, and thereby is involved in bone resorption.^23^ Our findings suggest that PTPN2 might protect bone resorption by recognizing CSF1R and dephosphorylating CSF1R at Y807 site. Nevertheless, further mechanistic studies are needed to reveal the role of Y807 dephosphorylation in the regulation of bone resorption.

In conclusion, to our knowledge this is the first study to demonstrate that PTPN2 dephosphorylates CSF1R at Y807 site and inhibits alveolar bone resorption in diabetic periodontitis mice. Our results suggest that PTPN2 and CSF1R are potential targets for the therapy of diabetic periodontitis or other bone loss‐related diseases.

## CONFLICT OF INTEREST

All authors declare no conflict of interest.

## AUTHOR CONTRIBUTIONS

XX designed the study, DZ, YJ, DS, ZZ, CZ, LD performed the experiments and analysed the data. All authors participated in writing the manuscript and approved the manuscript.

## Data Availability

Data are available upon request.
